# Prognostic significance of B7-H3 expression in patients with colorectal cancer: A meta-analysis

**DOI:** 10.12669/pjms.326.11511

**Published:** 2016

**Authors:** Heng Fan, Jian-hua Zhu, Xue-qing Yao

**Affiliations:** 1 Heng Fan, Department of Intensive Care Unit, Ningbo First Hospital, Ningbo, China, Department of General Surgery, Guangdong General Hospital & Guangdong Academy of Medical Sciences, Southern Medical University, Guangzhou, China; 2 Jian-hua Zhu, Department of Intensive Care Unit, Ningbo First Hospital, Ningbo, China; 3 Xue-qing Yao, Department of General Surgery, Guangdong General Hospital & Guangdong Academy of Medical Sciences, Southern Medical University, Guangzhou, China

**Keywords:** B7-H3, Colorectal cancer, Meta-analysis, Prognosis

## Abstract

**Objective:**

The co-stimulatory molecule B7-H3 plays an important role in prognosis of several malignancies. However, its prognostic value in clinic in patient with colorectal cancer (CRC) is still controversial. This meta-analysis evaluated the relationship between B7-H3 expression and the outcomes of CRC patients.

**Methods:**

PubMed, Google Scholar, Embase, CNKI and Wanfang database were searched for the studies on the relationship between the expression of B7-H3 and prognosis of CRC patients. Pooled odds ratios (ORs) analysis with 95% confidence interval (95% CIs) for lymph node metastasis, 24-month overall survival and 72-month overall survival were performed mainly using Review Manager 5.0.

**Results:**

Six articles including 1,202 total CRC cases were included for the meta-analysis. Pooled analysis with fixed-effects model showed that B7-H3 expression had no relationship with lymphatic metastasis in CRC patients (Fixed-effects, OR= 1.18; 95 % CI:0.87–1.61, P=0.28). However, B7-H3 expression was associated with 24-month overall survival (Fixed-effects, OR=0.48, 95% CI:0.32–0.74, P<0.001) and 72-month overall survival (Fixed-effects, OR = 0.61, 95% CI: 0.43–0.85, P< 0.01) in CRC patients.

**Conclusion:**

The co-stimulatory molecule B7-H3 expression is negatively associated with lymph node metastasis in CRC. However, B7-H3 detection might be a feasible and effective means to predict the prognosis in CRC patients.

## INTRODUCTION

Colorectal cancer (CRC) is the fourth most common malignancy leading to death globally, and accounts for about 600,000 deaths every year in the world.^1^ The countries of Europe, Oceania and North America are reported to have the higher morbidity, whereas some countries of Asia and Africa have comparatively lower morbidity.^2^ The prognosis of CRC patients has steadily increased during the past 10 years in most of countries. Five year survival has reached nearly 65% in high-income countries, such as the USA, European countries and Australia, but remains less than 50% in low-income countries.^3^

Lymph node Meta stasis is an important pathway of CRC cancer systemic metastasis, and is closely related to the prognosis of CRC patients.^4^ Frequently, regional lymph nodes are initial metastasis sites, and then tumor cells migrate into the lymph nodes.^5^,^6^ B7-H3 was first identified in 2001, is an immune-regulatory protein in B7 family of T cell co-stimulatory molecules, which may be a new prognostic marker.^7^,^8^ 2IgB7-H3 and 4IgB7-H3 are two isoforms in human beings.^9^ B7-H3, as a very important co-stimulatory molecule, promotes T-cells proliferation and induces T-cell receptor signaling interferon production.^10^ However, B7-H3 also serves as natural killer cells, T cell co inhibitor, and antigen presenting cells.^11^,^12^ Several subsequent studies support the viewpoint that B7-H3 inhibits the activation of T cell, and a stimulatory immunological role of B7-H3 in the area of antitumor immunity.^13^-^15^

Some researchers suggest that the B7-H3 signaling system is one of efficient pathway in regulating lymphangiogenesis.^5^,^6^,^16^ B7-H3 may show resistance to apoptosis via the signaling pathway of Jak2-STAT3, and facilitates vessel enlargement into the surrounding lymphatic vessels.^17^ Previous epidemiological studies have showed that B7-H3 over expression was correlated with lymphatic metastasis in CRC.^18^-^23^ However, the results remain inconclusive. Some studies have reported that B7-H3 high expression in CRC patients are associated with lymph node metastasis and prognosis value,^18^,^22^,^23^ while the other studies report contrary findings.^19^,^20^,^21^ To derive the precise relationship between B7-H3 expression and clinical prognosis in CRC patients, a meta-analysis of six independent studies based on a total of 1,202 CRC patients was performed, to see the relationship between B7-H3 expression and CRC prognosis.^24^-^26^

## METHODS

### Search strategy and inclusion criteria

A systematic literature search of Google Scholar, Embase, PubMed and two Chinese databases (Wanfang and Chinese National Knowledge Infrastructure database) were conducted by two study investigators independently for all related articles about the outcomes value of co-stimulatory molecule B7-H3 expression in CRC patients. The terms used in the research included “co-stimulatory”, “B7-H3”, “colorectal cancer”, “CRC”, “immunochemistry”, “colorectal neo plasma(s)”, “colorectal carcinoma”, “metastasis”, and “prognosis”. All eligible studies and their references were browsed, and no date or language limits were applied.

### Inclusion and exclusion criteria

The included studies in this meta-analysis were as per the following criteria:

1. Measures B7-H3 expression in the CRC tissue with immunohistochemistry (IHC)
2. Provides survival time information according to B7-H3 expression
3. Patients’ follow up time is more than 48 months.
  Exclusion criteria included:
  - Reviews, comments, Letters, meeting abstracts
  - Animal studies
  - Studies with incomplete and/or duplicate data.

### Data extraction

Two researcher`s extracted the full-text articles from each study independently. These full texts were then evaluated as per the inclusion criteria. The following information was collected: the title, author’s name, publication time, total number of cases, country, primary end point, follow-up time, and patients’ numbers with positive expression of B7-H3, etc. Any disagreement between the reviewers was resolved by consensus with a third researcher.

### Statistical analysis

All data was analyzed by Review Manager 5.0 program (RevMan 5.0; The Cochrane Collaboration 2008, Denmark) and Stata 12.0 (Stata Corporation, USA) software. *P* value level of heterogeneity test was <0.1, heterogeneity exists obviously, the pooled analysis was calculated by the random-effects model. Otherwise, a Fixed-effects model was used to calculate the merged data. The odds ratios (ORs) with 95% confidence interval (95% CIs) were calculated for all included studies. *P* value level < 0.05 was considered statistical significance. The heterogeneity was assessed by using *I*^2^ statistics, and publication bias was tested by Egger’s test and Begg’s funnel plot.

## RESULTS

### Literature search process

**Fig. 1:**
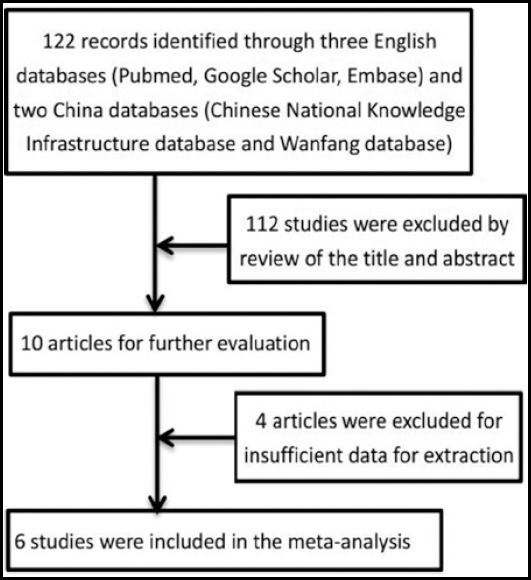
Flow diagram of process for identification of studies.

After a complete research based on the above mentioned criteria, a total of 122 suitable articles were reviewed. After reviewing the titles or abstracts, 112 articles were excluded because they were not relevant to the role of B7-H3 in CRC patients and also had insufficient data. Therefore, 10 papers were preliminarily identified for further evaluation. Among those 10 studies, six studies reported the data about the lymph node metastasis,^18^-^23^ including one Chinese article,^19^ and three studies reported the data about the B7-H3 expression on overall survival (Fig.1).^20^-^22^ Moreover, 1,202 cases provided lymphatic metastasis data, among which 452 cases were with metastasis and 745 cases without that, and 937 cases had positive B7-H3 expression and 265 cases were negative B7-H3 expression (Table-I).

**Table-I:** Summary of included studies.

Studies	Location	Journal	Study Design	Setting	B7-H3 Expression	Primary end point	Follow-up
Wang et al 2009^18^	China	Chin J Gen Surg	Retrospective	Single-center	71/80	NA	NA
Sun et al. 2010^19^	China	Cancer Immunol Immunother	Prospective	Single-center	54/102	NA	NA
Mao et al. 2013^20^	China	Chin Med J	Retrospective	Single-center	45/98	20-month OS	60-month OS
Ingebrigtsen et al.2014^21^	Norway	BMC Cancer	Prospective	Five centers	637/731	20-month OS	80-month OS
Bin et al.2014^22^	China	J Surg Res	Prospective	Single-center	59/104	48-month OS	192-month OS
Jiang et al. 2016^23^	China	Oncotarget	Prospective	Single-center	71/87	NA	NA

NA:not applicable; OS:overall survival.

### Pooled analysis results

**Fig. 2:**
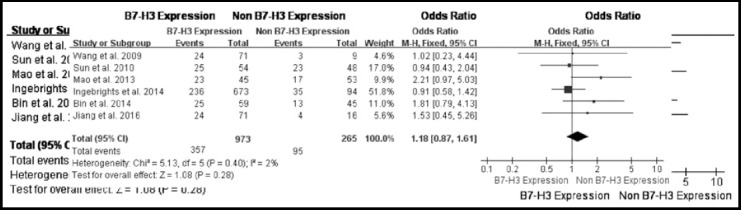
Forrest plot (Fixed-effects model) of odds ratios for the association of B7 H3 expression with lymph node metastasis in patients with CRC.

**Fig.3:**
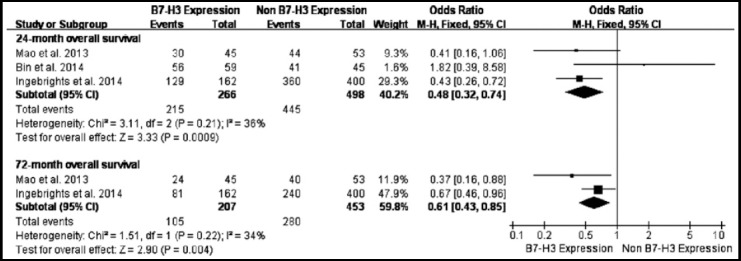
Forest plot (Fixed-effects model) for odds ratios of 24-month overall survival and 72-month overall survival.

The B7-H3 expression rate and cumulative metastasis rate of CRC were 77.95% (937/1202) and 37.6% (452/1202), respectively. The B7-H3 positive expression cases had a metastasis rate of 38.1% (357/937) that was slightly higher than 35.84% (95/265) in B7-H3 negative expression cases of six studies, there was no heterogeneity between studies (*I*^2^=2%, *P*=0.4). Fixed-effects model showed B7-H3 expression had no relationship with lymphatic metastasis in patients with CRC (Fixed-effects, OR=1.18; 95 % CI: 0.87–1.61, *P*=0.28) (Fig.2). Among these three studies with 933 CRC patients had the 24-month overall survival, there were no difference between study heterogeneities (*I*^2^=36%, *P*=0.21). Fixed-effects model showed the expression of B7-H3 was associated with 24-month overall survival in CRC patients (Fixed-effects, OR=0.48, 95% CI: 0.32–0.74, *P*<0.001) (Fig.3). Further, two studies with fixed-effects model showed the expression of B7-H3 was associated with 72-month overall survival in CRC patients (Fixed-effects, OR = 0.61, 95% CI: 0.43–0.85, *P*< 0.01) (Fig.3).

### Publication bias

**Fig.4:**
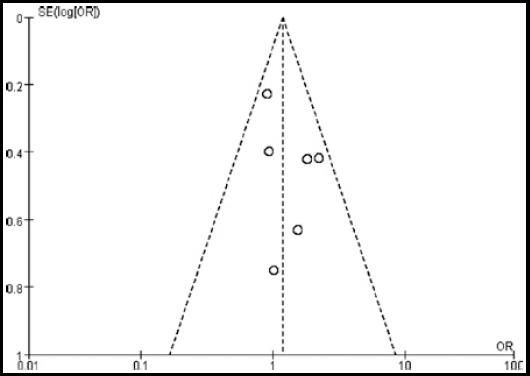
Begg’s funnel plot for publication bias test with pseudo 95% confidence limits for the studies related to lymph node metastasis.

Publication bias was evaluated by Egger’s test and Begg’s funnel plot. Funnel plots’ shape did not appear dissymmetry, and Egger’s test also showed that there was no publication bias in the association of B7-H3 expression with lymph node metastasis in CRC patients (Fig.4, P>0.05). Since there are no more than five prognostic studies about 24-month overall survival and 72-month overall survival, these studies’ publication bias was not performed.

**Table-II:** 24-month survival, 48-month survival and 72-month survival.

Name	Year	n	24-month survival		48-month survival		72-month survival	

			B7-H3 P	B7-H3 N	B7-H3 P	B7-H3 N	B7-H3 N	B7-H3 N
Wang et al^18^	2009	80	Null	Null	Null	Null	Null	Null
Sun et al^19^	2010	102	Null	Null	Null	Null	Null	Null
Mao et al^20^	2013	98	30/45	44/53	27/45	42/53	24/45	40/53
Ingebrigtsen et al^21^	2014	731	129/162	360/400	113/162	266/400	81/162	240/400
Bin et al^22^	2014	104	56/59	41/45	47/59	40/45	Null	Null
Jiang et al^23^	2016	87	Null	Null	Null	Null	Null	Null

B7-H3 P: The cases with B7-H3 expression positively. B7-H3 N: The cases with B7-H3 expression negatively.

## DISCUSSION

Colorectal cancer (CRC) is one of the most familiar malignancy types in the world, and its pathophysiology related with the interaction between individual genetic molecular background and the surrounding environmental factors.^27^-^29^ Studies have proved that genetic modification together with get toxenobiotics bacterial toxins, diet, cigarette smoke, and drugs may increase CRC risk.^30^ However, it remains unclear on the biological pathway contact life style characteristics and CRC.

Many investigators have studied the effects of the co-stimulatory molecule B7-H3 on the development of anti-apoptosis of CRC.^31^-^33^ There is no doubt that the B7 family is an important cancer player, thus the B7-H3 study will be very promising in the field of malignancy research. High expression of B7-H3 strengthened the resistance to chemotherapeutics and anti-apoptotic ability, nevertheless knockdown of B7-H3 might enhance the sensitivity of apoptosis induced by drug.^34^-^37^ Up to now, many studies had reported that the high-expression of B7-H3 significantly correlated with lymphatic metastasis and lymphangiogenesis in CRC.^18^,^22^,^23^ However, other reports did not prove such relationship, or opposite correlations were found.^19^,^20^,^21^ To date, there is no agreement on relationship between high-expression of B7-H3 detected by immunochemistry and survival in CRC patients. Accurate assessment of B7-H3 over expression impact on CRC patients is needed.

In CRC, the lymphatic system is always primarily metastasis pathway and lymphatic metastasis is a key prognostic role for the disease.^38^-^41^ Due to the limited sample size of individual studies, broad consensus on relationship between the over expression of B7-H3 and lymphatic metastasis in CRC has not reached yet. This meta-analysis including 1,202 cases from six published studies explored the relationship between the B7-H3 expression and prognosis of CRC patients. The overall results indicated that the expression of B7-H3 was not associated with lymph node metastasis. However, the B7-H3 expression in CRC patients is significantly associated with 24-month and 72-month overall survival, including three and two independent studies, respectively. The present studies’ data suggest that B7-H3 expression is significantly related with the outcome of CRC patients, and those patients with B7-H3 over expression may have poorer survival rate. Our findings will be very promising for the outcome and treatment strategy of CRC, in addition to improve pathophysiology understanding.

### Limitations of the study

Our results should be interpreted cautiously since there are a few limitations in the meta-analysis. Firstly, there were not enough data available from studies, and the total number of patients was only 1,202. Moreover, some clinical factors might lead to bias, such as age and/or chemotherapies plans in study. Secondly, our study might be overestimated; as there are two retrospective cohort studies included which might have higher risk of biases.^18^,^20^ Hence, larger and well-designed randomized controlled trial are needed to further assess the relationship between the B7-H3 over expression and CRC prognosis. Moreover, sufficient number of prospective studies are needed to further evaluate the accurate outcome effect of the B7-H3 expression in CRC patients.

This meta-analysis includes six studies which suggest that B7-H3 expression is not associated with lymphatic metastasis in CRC patients. However, the expression of B7-H3 might be an important outcome factor in CRC patients, if tested by immunochemistry. Moreover, considering the challenges relevant patient included and improvement in understanding of the pathophysiology mechanisms of B7-H3 may improve outcomes. As such further studies need to ascertain with larger Randomized Clinical Trials, widely accepted evaluation methods are necessary to expound the accurate outcome effect of the B7-H3 expression in CRC patients.
